# Medial temporal lobe atrophy is underreported and may have important clinical correlates in medical inpatients

**DOI:** 10.1186/s12877-015-0066-4

**Published:** 2015-06-16

**Authors:** Gustav Torisson, Danielle van Westen, Lars Stavenow, Lennart Minthon, Elisabet Londos

**Affiliations:** Department of Clinical Sciences, Clinical Memory Research Unit, Lund University, Lund, Sweden; Diagnostic Radiology, Clinical Sciences, Lund University, Lund, Sweden; Center for Medical Imaging and Physiology, Skåne University Hospital, Lund, Sweden; Department of Internal Medicine, Skåne University Hospital, Malmö, Sweden

**Keywords:** Dementia, Neurodegenerative diseases, Computerised tomography, Inpatients

## Abstract

**Background:**

The diagnostic workup in dementia includes brain imaging with reading focussed on signs of cerebrovascular and neurodegenerative disease. We hypothesised that these findings may be underreported in hospital patients, where imaging is often performed to rule out obvious pathology such as haemorrhage. In this study, we review cranial computed tomography (CT) in medical inpatients for white matter changes and atrophy. Our aim was to determine the clinical relevance of such findings and to what extent they were underreported.

**Methods:**

Records from 200 inpatients aged over 60 years, who had been subjected to MMSE (mini-mental state examination) and CDT (clock-drawing test), were reviewed for cranial CT. Transverse and coronal slices were reassessed using visual rating scales regarding white matter changes (WMC), global cortical atrophy (GCA) and medial temporal lobe atrophy (MTA). Findings were compared with the original radiology reports and cognitive test results.

**Results:**

Cranial CT had been performed in 94 of 200(47 %) patients. Of these, 58(62 %) had abnormal WMC, 35(37 %) abnormal GCA and 34(36 %) abnormal MTA. All three findings had associations with cognitive test results. Abnormal MTA was associated with lower results on the overall score on MMSE and on orientation, memory and language items. All three measurements were underreported in the original radiology reports; none of the 34 patients with abnormal MTA had been reported originally.

**Conclusions:**

Signs of neurodegenerative disease, especially MTA, were highly underreported in cranial CT scans performed in medical inpatients. At the same time, MTA seemed to hold the most important clinical correlates. Our results suggest that MTA should be reported more regularly in this setting.

## Background

The need for improved diagnosis rates in dementia is often emphasised [1, 2]. Today, one third of people in the United Kingdom will have dementia at the end of life, of which half will have been diagnosed [3, 4]. To improve diagnosis rates, policy-makers have turned their attention towards emergency hospitals, where dementia prevalence is higher than in community settings [5–9].

The diagnostic workup in dementia includes cognitive tests and brain imaging, using a CT scan or an MRI [10–13]. Brain imaging is used to exclude intracranial masses, to support clinical diagnosis and to differentiate between diseases causing the dementia syndrome [14]. For example, a diagnosis of Alzheimer’s disease (AD) is supported by medial temporal lobe atrophy (MTA), while white matter changes (WMC) or infarcts are prerequisites for vascular dementia (VaD) [15–17].

We hypothesised that brain imaging could be used to increase case-finding in a general hospital. Many elderly inpatients undergo cranial CT, often with a low clinical yield [18]. However, standardised assessments of white matter changes and atrophy are usually not done, even in patients with cognitive impairment [19]. In a previous publication, we found that 73 % of a group of 200 medical inpatients had abnormal results on cognitive tests, of whom only 8 % were previously diagnosed [20]. In the present study, we reviewed routine cranial CT in this group, focussing on white matter changes and atrophy. We assumed that these findings would be underreported, as CT would primarily have been done to rule out infarcts and haemorrhage. To assess the clinical relevance of the radiological findings, they were compared with cognitive tests.

## Methods

The study was carried out at the Department of Internal Medicine at Skåne University Hospital, a 700-bed tertiary care facility in Malmö, Sweden. The present study is a secondary analysis based on a previously published intervention study, comprised of 200 patients seeking emergency medical care for a wide variety of presenting complaints. The inclusion has previously been described in detail [21]. Included patients were over 60 years old, living at home and had capability to perform crude cognitive tests. Patients with terminal disease, language barrier, deafness, aphasia, blindness and severe disease associated with inability to communicate were excluded. All patients in the original study who had undergone a routine clinical CT were eligible for the present study.

### Measurements

All data, except for the review of CT scans, have been prospectively collected in a standardised way. Cognitive tests were carried out at the wards, in private, by three experienced testers who had received special training. Cognitive tests were carried out when the patients had been stabilised, patients with fever (>38 °C), electrolyte imbalance, anaemia or elevated C-reactive protein (>50 mg/L) were not tested until the condition had subsided.

The mini-mental state examination (MMSE) was used [22]. The maximal score is 30 points; a cut-off of < 24 points is often used to signify cognitive impairment in medical inpatients. MMSE consists of ten items: orientation, registration, attention, recall, naming of objects, repetition, 3-step command, reading, writing and figure copying. The typical result in AD is low scores on orientation and recall, abilities that are dependent on the temporal lobe. In VaD the scores differ depending on the areas involved but typically attention and executive functions are affected [23].

We also used the clock-drawing test (CDT). The patient was instructed to “draw the face of a clock and set the time at ten past eleven”. Points from 0 (worst) to 5 (best) were given, according to Shulman, with a score < 4 points considered abnormal [24]. The CDT tests executive ability and planning, subcortical functions and functions of the frontal lobe.

Comorbidity was determined using interviews with patients and proxies as well as review of medical records. Any conditions from interviews or diagnoses noted in the medical record during the present or three preceding hospitalisations were recorded. Combined comorbidity was obtained using the Charlson comorbidity index [25].

### Ethics statement

The study was performed according to the declaration of Helsinki and all patients have given their written informed consent. The original study and the secondary analysis have both been approved by the regional ethics committee at Lund University.

### Brain imaging

No brain imaging was planned originally and therefore routine CT scans were reviewed retrospectively for the present study, regardless of indication. In dementia research, MRI has primarily been used in order to grade white matter changes and atrophy. However, when simple visual rating scales are used, Wattjes et al. have shown that results from CT and MRI are comparable [26].

We searched the hospital’s digital picture archiving and communication system (PACS) for CT scans performed ± 1 year from the admission in the original study. If several CT had been performed, the one closest in time to cognitive testing was selected for review. All patients in whom CT was performed, except one, had been imaged at the radiology department at Skåne university hospital in Malmö, the remaining patient underwent CT at the radiology department at the same hospital, now in Lund. All CT examinations were performed according to routine clinical protocols at 16–64 detector row equipment with automatic dose modulation. In all cases, raw data were reconstructed in the axial (4.5 mm slices in Malmö, 5 mm slices in Lund) and coronal (3 mm slices) plane. Raw data as well as reconstructions were saved at the hospital’s PACS system. Patient information including the referral form is simultaneously available by the radiological information system. For each CT scan, the date and referring department was retrieved. We also recorded if any symptoms of cognitive impairment (“dementia”, “confusion”, “delirium”, “memory impairment”) were mentioned in the referral form.

### Visual rating scales

The images were reviewed by an experienced neuroradiologist (DvW), who was blinded to all data except the referral form. Three visual rating scales were used, see below.

### White matter changes (WMC)

White matter changes are assumed to result from inadequate perfusion and are characterized histopathologically by enlarged perivascular spaces, demyelination and gliosis. In this study, WMC were scored in the axial plane according to a modification of the four-point scale developed by Fazekas et al. [27]. This scale has shown good to excellent inter-rater reliability and good correlation with volumetry [28]. The whole brain was rated as:

- 0: None or single punctate lesion
- 1: Multiple punctate lesions
- 2: Beginning confluency of lesions (bridging)
- 3: Large confluent lesions

Scores were dichotomised into normal (0–1 points) or abnormal (2–3 points) as a score of 1 point may well represent normal ageing [29, 30]. Also, the detection of punctate lesions is unreliable on CT except perhaps for the insula and thus a cut off of ≥ 2 was decided reasonable.

### Global cortical atrophy (GCA)

Global cortical atrophy represents the mean volume loss of the cerebral cortex as a whole. In this context, the GCA rating scale is used to give an overall estimate of atrophy without regional bias. The GCA rating was based on axial projections based using a four-point scale [31]. This scale has been shown to have a lower good inter- and intra-rater reliability [32]. Global atrophy was rated as:

- 0: No cortical atrophy
- 1: Mild atrophy (opening of sulci)
- 2: Moderate atrophy (volume loss of gyri)
- 3: Severe atrophy “knife blade atrophy”

The scores were dichotomised into normal (0–1 points) or abnormal (2–3 points) as a score of 1 point could represent normal aging [33].

### Medial temporal lobe atrophy (MTA)

Medial temporal lobe atrophy represents loss of volume in the hippocampal area. MTA is sensitive for Alzheimer’s disease but not specific; it can be found in other dementias as well [34]. In prospective studies in non-demented subjects, MTA has been shown to predict future dementia in general and Alzheimer’s disease in particular [35, 36]. The Scheltens scale was used to rate MTA, this scale has excellent intra-rater and inter-rater reliability and significant correlations with volumetry [37–40].

- 0: No atrophy
- 1: Widening of choroid fissure
- 2: As 1 + widening of temporal horn of lateral ventricle
- 3: As 2 + lowered height of hippocampal formation
- 4: As 3 + further volume loss of hippocampus.

**Fig. 1:**
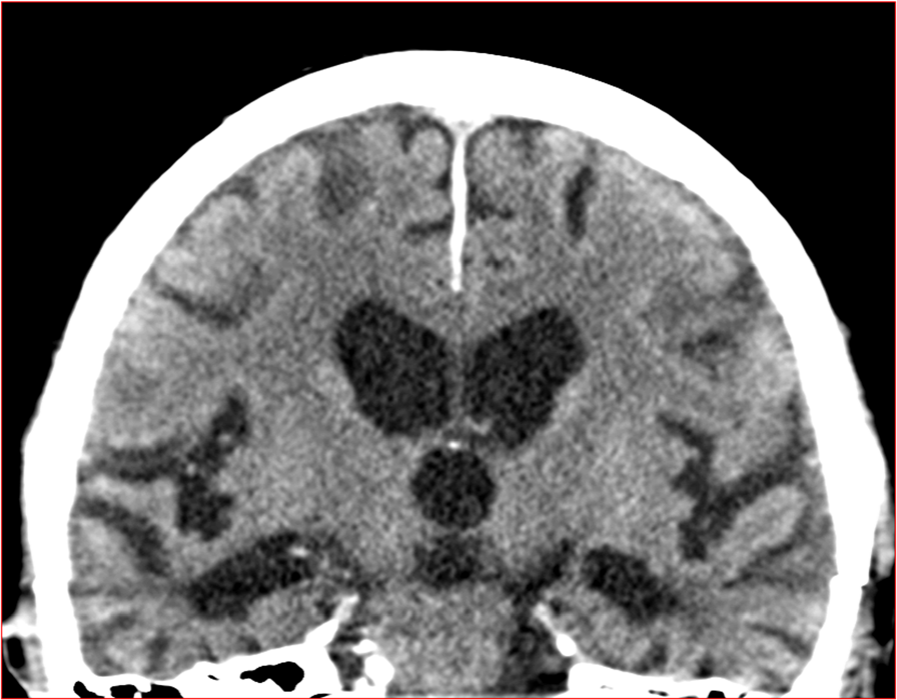
Medial temporal lobe atrophy. Example of abnormal medial temporal lobe atrophy in a CT scan in a study patient representing a score of 3 on the left side and 4 on the right side. This was not mentioned in the original report. This patient had an MMSE score of 22 points with 0 points on the memory item. This patient had noprevious mentioning of cognitive impairment in medical records

The scores were dichotomised into normal (0–1 if age < 75, 0–2 if age ≥ 75) and abnormal (2–4 if age < 75, 3–4 if age ≥ 75) as a certain amount of MTA occurs in normal aging [41, 42]. If there was a discrepancy between left and right side, the highest degree of atrophy was used. An example of MTA is illustrated in Fig. 1.

### Original radiology reports

The original radiology reports were examined thoroughly regarding any description of white matter changes, among these “small vessel disease”, “leukoaraiosis”, “degenerative vascular change”, as well as general atrophy and medial temporal lobe atrophy. For all three measurements, the same scoring was used.

- NA: Finding not mentioned in original report
- 0: Finding excluded in original report.
- 1: Finding described as “mild” or not quantified
- 2: Finding described as “moderate”
- 3: Finding described as “severe”

### Statistical method

We compared the patients who had undergone CT scans with the ones who had not regarding baseline characteristics. Student’s t-tests, chi-square tests and Mann–Whitney U-tests were applied where appropriate. A subgroup analysis was done for the patients whose referrals had mentioned cognitive impairment.

To obtain an estimate of intra-rater reliability, 30 CT scans were re-rated by the same rater, blinded to the previous rating, with 12 months between the ratings. Intra-rater reliability was expressed with weighted Kappa values.

The association between cognitive tests and the dichotomised radiological measurements was studied using Mann–Whitney U-tests. On MMSE, the total score as well as the different items were compared. In addition, the three measurements were compared to cognitive impairment regarding sensitivity, specificity and predictive values. Cognitive impairment was defined as having at least one abnormal cognitive test result (MMSE < 24 or CDT < 4).

All calculations were done using SPSS version 20.0. A two-sided p value of < 0.05 was considered significant.

## Results

**Fig. 2:**
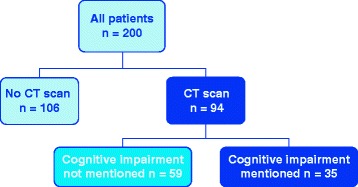
Outline of the study. The original study comprised of 200 patients. Of these, 94 had performed a CT scan. In 35 of these, cognitive impairment was mentioned in the referral

In total, 94 of 200 patients (47 %) had undergone CT ± 1 year of cognitive tests. Of these, 26 were scanned before, 44 during and 24 after the index hospitalisation. The median interval between CT and cognitive testing was 12 days (interquartile range 1–132 days). The referral for CT was issued by the ED or a hospital ward in 87 patients, by outpatient clinics at the hospital in 5 patients and by GPs in 2 patients. Cognitive impairment was mentioned in 35/94 (37 %) of referrals, see Fig. 2.

The patients who had undergone a CT scan had a higher prevalence of hypertension and stroke. This group was also more cognitively impaired; baseline data are displayed in Table 1.

**Table 1:** Baseline data

Characteristic	No CT scan n = 106	CT scan n = 94	p value
Age	83.8 (8.3)	83.0 (8.0)	0.46
Female sex	63 %	67 %	0.66
Intervention in original study	55 %	44 %	0.12
Living alone	62 %	72 %	0.14
Ischemic Heart Disease	38 %	29 %	0.23
Heart Failure	30 %	26 %	0.53
Hypertension	42 %	56 %	0.047
COPD	24 %	15 %	0.15
Stroke/TIA	10 %	31 %	<0.001
Diabetes	22 %	25 %	0.74
Cancer, nonskin	26 %	31 %	0.43
Diagnosed with dementia or MCI	6 %	10 %	0.42
Charlson comorbidity index	2.2 (1.4)	2.3 (1.6)	0.70
MMSE	23.9 (3.7)	21.6 (4.4)	<0.001
CDT	3.8 (1.1)	3.0 (1.2)	<0.001

All data are presented as mean (SD) or percentages. Abbreviations: MCI = mild cognitive impairment, COPD = chronic obstructive pulmonary disorder, TIA = transient ischemic attack, MMSE = mini-mental state examination

**Fig. 3:**
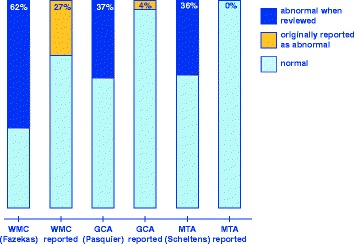
Reporting of findings. Chart showing the reporting frequency of abnormal findings next to the reviewed findings. WMC = white matter changes, GCA = global cortical atrophy, MTA = medial temporal lobe atrophy

All three measurements were highly prevalent and underreported in the original reports, see Table 2 for exact scores and Fig. 3 for dichotomised values. Intra-rater weighted Kappa values were good to excellent, with 0.90 for WMC, 0.77 for GCA, 0.94 for MTA (left) and 0.97 for MTA (right).

**Table 2:** Visual rating scales (n = 94)

White matter changes (WMC)	Original report	Review
0 points	9	17
1 points	25	19
2 points	14	31
3 points	11	27
WMC not commented	35 (37 %)	-
Global cortical atrophy (GCA)		
0 points	8	15
1 points	22	44
2 points	4	30
3 points	0	5
GCA not commented	60 (64 %)	-
Medial temporal lobe atrophy (MTA)		
0 points	4	14
1 points	4	26
2 points	0	25
3 points	0	22
4 points	NA	7
MTA not commented	86 (91 %)	-

Table 2. Frequency of the radiological findings as described in the original report and on review. The original reports were rated 0 (finding negated), 1 (mild), 2 (moderate), 3 (severe). For the review scores, the Fazekas, Pasquier and Scheltens scales were used for WMC, GCA and MTA respectively.

NA: not applicable since the original reports were rated from 0 to 3

When cognitive impairment was mentioned in the referral, WMC reporting increased in the original radiology reports. Upon review, abnormal MTA was more prevalent in this subgroup, see Table 3.

**Table 3:** Analysis of the subgroup in which cognitive impairment was mentioned in the referral

Characteristic	CI not mentioned n = 59	CI mentioned n = 35	p value
Age	83.6 (8.3)	81.8 (7.3)	0.27
Female sex	61 %	77 %	0.12
In intervention originally	44 %	43 %	1.00
Living alone	66 %	83 %	0.10
Charlson index	2.2 (1.5)	2.5 (1.7)	0.45
MMSE	22.5 (4.2)	20.1 (4.4)	0.009
Clock-drawing test	3.1 (1.2)	2.7 (1.3)	0.10
WMC not commented in original report	27 (46 %)	8 (23 %)	0.03
GCA not commented in original report	40 (68 %)	20 (57 %)	0.38
MTA not commented in original report	54 (91 %)	32 (91 %)	1.00
WMC abnormal on review	34 (58 %)	24 (69 %)	0.38
GCA abnormal on review	20 (34 %)	15 (43 %)	0.51
MTA abnormal on review	16 (27 %)	18 (51 %)	0.03

Data are presented as mean (SD) or number (percentages). Abbreviations: CI = Cognitive Impairment, MMSE = mini-mental state examination.

### Visual rating scales vs. cognitive tests

Abnormal WMC was associated with lower scores on the MMSE item of repetition (p = 0.012). Abnormal GCA was associated with lower scores on MMSE total (p = 0.009), orientation (p = 0.04), 3 step command (p = 0.010), figure copying (p = 0.03) and on clock-drawing test (p = 0.02). Abnormal MTA was associated with lower scores on MMSE total (p = 0.006), orientation (p = 0.03), recall (p < 0.001) and reading (p = 0.018). When compared to cognitive impairment in general, the sensitivity, specificity and predictive values are shown in Table 4.

**Table 4:** Relationship with cognitive impairment

Property	WMC	GCA	MTA
sensitivity	68 %	40 %	39 %
specificity	79 %	71 %	79 %
positive predictive value	92 %	91 %	91 %
negative predictive value	28 %	18 %	18 %

Properties of abnormal results on the three visual rating scales when compared to cognitive impairment, defined as having at least one abnormal cognitive test result (MMSE < 24 points or CDT < 4 points)

### Other radiology findings

Thirty-two patients had other findings, apart from WMC, GCA and MTA, upon review. Thirty of these were found to have cerebral infarctions/haemorrhage in the parenchyma. The white matter changes attributed to infarct areas were not included in the rating of WMC. One patient had a small residue of a previous subdural haematoma and one patient had (<5 mm) bilateral hygromas. When dichotomised into absent/present, other findings were not associated with lower scores on MMSE or CDT (p = 0.41 and p = 0.59, respectively)

## Discussion

In this study, WMC, GCA and MTA were rated in cranial CT:s of a general hospital population. All three measures were found to be highly prevalent, underreported and had seemingly important clinical correlates. This held especially for MTA, in spite of MTA being easy to assess on routine CT scans that include coronal reconstruction.

The underreporting of MTA may have several reasons. Firstly, emergency (<24 hours) cranial CT in the non-trauma population is performed on a wide variety of indications in search of findings requiring immediate attention. Thus, referrals often inquire for signs of acute ischemia, haemorrhage, and other conditions. Hypothetically, the radiologist is likely to reply to these specific questions without paying attention to findings related to chronic disease, such as MTA. However, radiological reports even for ED referrals frequently mention findings such as hypertrophic sinonasal mucosa in patients where the clinical significance of such finding likely is low [43]. Also, as in this study, WMC are quite often reported, albeit having little or no clinical significance in the acute situation. Thus, habits among radiologists seem to influence reporting.

Secondly, MTA could be underreported since radiologists may not have been trained to look for it and grade its severity. For example, the radiology reporting initiative that the Radiological Society of North America brought forward in 2012 does not mention atrophy in its template report and the ventricular system can only be graded as small or large (http://www.radreport.org/txt/0000004).

Thirdly, there may be a hesitation in reporting findings related to cognitive impairment when the referral lacks such information. However, MTA is unspecific and occurs in normal ageing as well as in small vessel disease without cognitive impairment. Thus, mentioning MTA does not imply a diagnosis of neurodegenerative disease and may be performed in all patients. In addition, our results show that when the referral did in fact include information on cognitive impairment, only reporting of WMC increased, not of MTA. Again, this suggests that habits influence reporting rather than concern of overstating findings associated with neurocognitive disorders. However, our results cannot justify the apparent habit of reporting WMC more than MTA

GCA was also underreported, however not graded; GCA is an unspecific finding, not connected to any specific disease, and possibly less reproducible and more prone to inter- and intra-rater variation. In addition, the cut-off for normalcy is not as well documented as for MTA; most cognitively impaired as well as unimpaired elderly may be graded as 1 or 2, leaving grade 3 only as definitely pathological. However, grade 3 is not often encountered. The clinical importance of GCA is probably restricted to longitudinal studies, such as for the evaluation of long-term effects of stroke and trauma; however GCA is reported here for completeness.

Regarding clinical associations, all three abnormal radiological findings had high positive predictive values when compared to cognitive tests. If a CT had been done, an abnormal finding would be associated with a 90 % risk of having an abnormal cognitive test. Sensitivity and specificity for the three tests were lower; however, no-one would recommend performing CT in all medical inpatients in order to detect cognitive impairment. Rather, our results suggest that abnormal findings should be reported *when* a CT has been done, regardless of indication.

Among the three measurements, WMC had the weakest associations with cognitive tests, with the MMSE item of repetition alone. Previous studies have shown that when atrophy and WMC co-exist, the importance of WMC is reduced, this could be an explanation why WMC had such a small impact [44] Abnormal GCA had several associations with MMSE items and with the CDT, representing a global impairment. Abnormal MTA had the strongest association, with memory impairment; this association was the only one that would sustain a conservative Bonferroni correction. In addition, MTA was associated with lower scores on MMSE total, orientation and language items. Together, this profile indicates a possible clinical presentation of AD, which would be supported by the finding of MTA. This is clinically important as there is symptomatic treatment for AD.

This study has several methodological issues related to its retrospective design:

Firstly, there is a possibility that the original intervention study may have affected the results, as the interventions acknowledged cognitive impairment [21]. If so, any bias would probably be towards increased reporting of cognitive impairment, as more attention was being paid to these symptoms. Secondly, there was a risk for selection bias as brain imaging was not planned originally and only patients who had undergone a CT could be reviewed. Therefore the findings regarding prevalence of WMC, GCA and MTA may be overestimated and should be interpreted cautiously.

Regarding the reliability of the CT scans, it is a weakness that only one person performed the visual ratings. However, the rater was a highly experienced neuroradiologist and the CT scans were performed on state-of-the-art equipment using coronal reconstructions. Regarding MTA, which is the focus of this paper, it has been shown to be the visual rating scale with the highest inter-rater reliability, with Kappa ranging from 0.82 – 0.91 in recent studies including coronal reconstructions [38, 40] These studies also show excellent intra-rater reliability for MTA, consistent with our results.

The validity of CT scans could be affected by the time gap between CT and cognitive tests. Cognitive impairment could have been due to delirium and non-existent at the time of CT. However, white matter changes and atrophy represent long-term processes in the brain parenchyma. With a median time between scan and cognitive tests of 12 days, the scans were considered reasonably accurate.

To the best of our knowledge, this is the first study to apply standardised visual ratings scales regarding WMC, GCA and MTA on routine CT in a general hospital population. The findings regarding prevalence and reporting frequency need to be replicated at another location to determine external validity. Most likely, this has to be done in a retrospective way to avoid bias from awareness. The results regarding clinical usefulness are promising, especially considering the heterogeneity of this population compared to memory clinic populations. These should be replicated in a prospective study.

Throughout this study, a special emphasis has been put on MTA. One reason for this was that MTA was the most underreported finding. Another reason was that MTA combines a high reliability with important clinical correlates in a way that the other two scales do not. As both clinicians and radiologists in this setting are probably unfamiliar with all of these visual rating scales, MTA should probably be emphasised first. Reporting of MTA could increase the yield of cranial CT in this population by improved case-finding in cognitive impairment and dementia. In addition, it could possibly contribute to differential diagnosis, with abnormal MTA being associated with an AD-like profile on cognitive tests.

## Conclusions

We propose that MTA should be reported more regularly on cranial CT in the general hospital setting. Given the need for improved case-finding in dementia, we suggest that abnormal MTA should be used as a red flag to signal to the clinician that this patient should be offered a workup regarding cognitive impairment.

## Abbreviations

CT: Computed tomography
MMSE: Mini-mental state examination
CDT: Clock-drawing test
WMC: White matter changes
GCA: Global cortical atrophy
MTA: Medial temporal lobe atrophy
MRI: Magnetic resonance imaging
AD: Alzheimer’s disease
VaD: Vascular dementia
GP: General practitioner
PACS: Picture archiving and communication system
ED: Emergency department
